# Do self-rated health and previous vaccine uptake influence the willingness to accept MPOX vaccine during a public health emergency of concern? A cross-sectional study

**DOI:** 10.1371/journal.pgph.0003564

**Published:** 2024-08-15

**Authors:** Joseph Asumah Braimah, Meshack Achore, Florence Dery, Martin A. Ayanore, Elijah Bisung, Vincent Kuuire

**Affiliations:** 1 Department of Public Health, St. Lawrence University, Canton, New York, United States of America; 2 Department of Population Health, Hofstra University, Hempstead, New York, United States of America; 3 Department of Geography, Geomatics & Environment, University of Toronto, Mississauga, Ontario, Canada; 4 Department of Health Policy Planning and Management, University of Health and Allied Sciences, Ho, Volta Region, Ghana; 5 School of Kinesiology and Health Studies, Queen’s University, Kingston, Ontario, Canada; PLOS: Public Library of Science, UNITED STATES OF AMERICA

## Abstract

Monkeypox (MPOX) was declared a global public health emergency of international concern in July 2022. Vaccinations may be an essential strategy to prevent MPOX infections and reduce their impact on populations, especially among at-risk populations. However, less is known about the factors associated with people’s willingness to accept the MPOX vaccine in resource-constrained settings. In this study, we examine the associations between self-rated health, previous vaccine uptake, and people’s willingness to accept the MPOX vaccine using cross-sectional data from four major cities in Ghana. The data were analyzed using descriptive and logistic regression techniques. We found that the acceptance of the MPOX vaccine is generally low (approximately 32%) in Ghana. The regression analysis reveals that individuals who did not receive vaccines in the past are much less likely to get the MPOX vaccine (AOR:.28; 95% CI:.62–2.37). The association between self-rated health and vaccine acceptance (AOR: 1.22; 95% CI:.62–2.37) disappeared after we accounted for covariates. Based on these findings, we conclude that vaccine uptake history may be critical to people’s uptake of the MPOX vaccine.

## Introduction

Epidemiological data on the recent monkeypox (MPOX) outbreak indicate that as of August 2023, 989,308 people contracted the disease, which resulted in 152 deaths across the six World Health Organization (WHO) regions [1]. The WHO African region accounts for 2.1% (1,902) of confirmed cases and 13% of deaths globally [1]. Although low, the disproportionally high mortality rate of MPOX in Africa reflects the region’s vulnerability to the disease and, indeed, to several other infectious diseases, underscoring the need for public health programs to curtail infections. In the specific case of Ghana, although MPOX infection is relatively low (127 confirmed cases), it is labelled as a high-risk country, hence the need for evidence-informed public health interventions [1, 2].

Vaccinations are recommended to prevent and reduce the spread of MPOX, especially for at-risk populations such as health workers, people with multiple sex partners, sex workers, and men who have sex with men [3, 4]. Vaccinations may also help reduce hospitalizations from the disease and lessen the burden on health infrastructure. Yet, in Ghana and most countries in sub-Saharan Africa, vaccine apathy and hesitancy are high, which can have adverse public health implications [4, 5]. This is particularly concerning as these contexts are characterized by weak health systems and socio-cultural practices such as elaborate funerals and weddings, which tend to facilitate the spread of infectious diseases such as MPOX [6, 7].

According to current research, people’s self-rated health may impact their vaccine uptake [8]. Research has mixed results examining the association between self-rated health and vaccine acceptance. For instance, in Ethiopia, it was observed that government healthcare workers with no known chronic health condition were more likely to receive the COVID-19 vaccine [9]. Individuals with poor self-rated health in Canada were less likely to be vaccinated against COVID-19 [10]. The authors argue that individuals with poor self-rated health may be concerned about the safety of available vaccines and, hence, less likely to accept them. On the contrary, O’Halloran et al. [11], in a study on influenza vaccination among high-risk populations in the United States, showed that individuals who perceive themselves as “healthy” are less likely to participate in preventive health activities, including influenza immunization [11].

Individual’s vaccination history may also impact their willingness to receive vaccines [5, 12, 13]. For example, in China, the history of influenza vaccination was associated with women’s acceptance of the influenza vaccine during the COVID-19 pandemic [14]. Beyond these factors, the literature also links people’s willingness to accept vaccines to sociodemographic and economic factors such as level of education, age, gender, and place of residence [5, 10, 13]. Likewise, knowledge of infectious diseases can also influence vaccine uptake reactions as it empowers them to make (un)informed health decisions [13–15].

Notwithstanding these studies’ usefulness in knowledge enhancement and public health programming, little is known about the relationships between self-rated health, vaccine uptake history, and people’s willingness to take the MPOX vaccine in Ghana [2, 5]. A critical study by Ghazy and colleagues [5] drew on cross-sectional data from social media platforms to examine MPOX vaccine acceptance among people in Ghana [5]. Their study used a snowball sampling technique and was limited to people (n = 550) with access to the internet and smartphones, which has implications for their findings.

Building on this earlier study, we examine the associations between self-rated health, history of vaccine uptake, and people’s willingness to accept the MPOX vaccine in Ghana using cross-sectional data from four major cities in Ghana. Understanding the relationship between self-rated health, vaccination history, and willingness to accept MPOX vaccinations can help develop appropriate interventions to improve vaccine uptake, especially for people in low- and middle-income countries.

## Materials and methods

### Ethics

The study received approval from the Queen’s University General Research Board and the University of Toronto Social Sciences, Humanities and Education Research Ethics Board. Participants in the study were asked for verbal consent. We did not use name identifiers in data analysis or presentation in order to protect individuals’ privacy and identities.

### Study design and data collection

This is a cross-sectional study. Survey data for this study was collected from October 2022 to October 2023. The survey was administered to households in four administrative cities in Ghana (Accra, Kumasi, Tamale, and Wa). We employed Krejci and Morgan’s [16] formula for estimating sample size based on the known population of each city. The sample was selected using a simple random sampling technique. A starting point was chosen using a random number generator, and every fifth house was interviewed thereafter. This was an in-person survey of 2059 household members using a Qualtrics-embedded tablet. The questionnaire was first piloted with 1% of the targeted households to verify face validity. Four research assistants proficient in English and the dominant language spoken in the research areas (Twi, Ga, Dagbani, and Dagaare) conducted the survey. The survey was initially written in English before being translated into the regional languages and back-translated into English. Data collected include the participant’s demographics, previous vaccine uptake, knowledge of the MPOX vaccine, health status, and use of preventive services. Recruitment of the participants was based on the following inclusion criteria: (a) adults 18 years or older and capable of participating in the study; (b) ability to communicate in English and/or the local language widely spoken in the study areas; and (c) willingness to participate.

## Measures

### Dependent variable

#### Willingness to receive the MPOX vaccine

The primary outcome variable, willingness to receive the MPOX vaccine, was measured by asking participants if they were willing to receive the MPOX vaccine. Participants are classified as willing to receive the vaccine if they answered yes to the question and unwilling if they answered no. The yes and no were further classified as 0 for the event and 1 for the no event.

### Independent variable(s)

#### Self-rated health

Self-rated health was determined by asking participants how they would rate their general health. Specifically, participants were asked, “How would you rate your personal health?”. The plausible response categories included “excellent,” “very good,” “fair,” and “poor.” These were then dichotomized, with “excellent” and “very good” categorized as “excellent” health and “fair” and “poor” categorized as “poor” health to simplify interpretation and discussion. Excellent and poor health were coded as yes and no, respectively. The categories are in quotes because we recognize that neither excellent nor poor health exists.

#### Previous vaccine uptake

Previous vaccine uptake was measured by asking participants if they “had previously willingly taken any vaccines (e.g., COVID vaccine) in the past year. Participants responded yes or no to the question. These responses (yes and no) were coded as 1 and 2, respectively, for the analysis.

#### Covariate

Covariates were selected following a literature review for variables previously identified as risk factors for the outcome variable of interest and linked with the exposure of interest. Specifically, variables were added to the model and controlled for if they were deemed confounders. The demographic covariates of interest for this study include age, gender, employment status, marital status, and health insurance status [17–19]. Age was measured by asking participants, “What is your current age?”. Participants indicated their age, which was recategorized into three groups (i.e., ages <30, 31–40, and >40). We measured gender by asking participants what their gender was. The responses (i.e., males and females) were assigned 1 and 2, respectively. Similar measures were applied to employment, marital, and health insurance status. Participants responded to predefined categories for these three variables. For employment status, participants indicated if they were employed or unemployed and were assigned 1 and 2, respectively. For marital status, participants indicated if they were married, in a common law partnership, single, or divorced. These were recategorized into married (i.e., married and common law) and single (i.e., single and divorced) and assigned 1 and 2, respectively. Health insurance status was measured by asking participants if they had health insurance. The responses were yes and no, and assigned 1 and 2, respectively. The study also controlled for knowledge of the MPOX vaccine. These knowledge questions included “Can people be infected with MPOX through bodily fluids"? “Can people reduce their risk of getting MPOX by using condoms”? Can people reduce their risk of getting MPOX by having one sexual partner”? These questions elicited yes or no responses, coded as 1 and 2, respectively. These dichotomous responses were converted into a continuous variable by adding the responses to all the knowledge questions. Lower values denote high knowledge about MPOX, and higher values denote less knowledge.

#### Analysis

All the statistical analyses were performed using STATA version 18 and SPSS version 24. The assumptions tested include (1) categorical dependent variables, (2) independence of observations, (3) outliers, and (4) whether or not the continuous independent variables are linearly associated with the log odds. Before conducting these analyses, we screened all explanatory variables, including the potential confounding variables, using univariable logistic regression analysis. Variables with a significance alpha less than or equal to 0.05 (α ≤ 0.05) from the univariate analysis were included in the multivariate regression model (model 3). We also assessed pairwise correlations among the independent variables using Spearman’s correlation, where |r|>0.7 is considered highly correlated. We also assessed outliers of the continuous variables using the cumulative distribution function of chi-square. A value of < 0.001 is considered an outlier. Furthermore, a binary logistics regression assessed the interactions between variables and their natural logs. With a p-value of < 0.05, interaction terms were considered a violation of assumption 4. Our analysis indicated that none of the logistic regression assumptions were violated.

After all assumptions were met, we developed three logistic regression models to explore the associations. Frequencies and their corresponding percentages were reported for the discrete variables. The weighted mean and standard deviation were produced and reported for continuous variables. Bivariate logistic regression was used to compute the unadjusted odds ratios (UOR) for model 1, which looked at the relationship between self-rated health and willingness to accept the MPOX vaccine, and model 2, which examined the relationship between previous vaccination status and willingness to accept the MPOX vaccine. Multivariate logistic regression was used to generate model 3, which adjusted for other variables (see model 3) in testing associations in models 1 and 2. The statistical significance was tested using 95% confidence intervals (95% CI).

We also assessed confounders and retained them in the model by calculating the magnitude of confounding (MoC), which is the percentage change in the unadjusted (crude) and adjusted measure of association (coefficient) of the variables of interest. We judged a variable as a confounder if the MoC is ≥ 10%. The multicollinearity was checked using the correlation coefficients of predictor variables. A correlation > 0.8 among variables indicates collinearity. The current model (model 3) returned the lowest Akaike’s Information Criterion (AIC) of 687.81 and a Bayesian Information Criteria (BIC) of 847.72. Additionally, we evaluated the model’s capacity to distinguish between outcomes of interest using the receiver operating characteristics (ROC) curve and the area under the curve (AUC). Model 3 returned an acceptable AUC of 0.78 (see Appendix A of S1 Text). Missing data were excluded from these analyses.

## Results

### Characteristics of the study sample

Characteristics of the 2059 study sample are presented in Table 1. Women made up 40.18% of the total sample. About 36.96% of the sample were educated, and 63.04% were married. Most (53.67%) of the participants were employed, and 63.42% were insured. Most (91.26%) participants rated their health as “excellent.” Regarding vaccination history, 41.75% were vaccinated in the past year, 65.75% were not, and 3.83% were hesitant to answer. Furthermore, 31.93% of participants said they were willing to receive the MPOX vaccine.

**Table 1:** Descriptive statistics of the study sample.

Variables		Frequency (%)
**Discrete variables**		
**Demographics**		
Self-rated health	Excellent	1,879(91.26%)
	Poor	180(8.74%)
Age	< 30	988(47.98%)
	31–40	502(24.38%)
	>40	569(27.63%)
Employment status	Employed	1,105(53.67%)
	Unemployed	954(46.33%)
Gender	Male	810(40.18%)
	Female	1,206(59.82%)
Average monthly income	<ghc2000	1,372(66.63%)
	Ghc 2001-ghc4000	241(11.70%)
	>4000	446(21.66%)
Level of education	No formal education	403(19.57%)
	Educated	1,656(80.43%)
Marital status	Single	761(36.96%)
	Married	1,298(63.04%)
Health insurance	Yes, insured	1,281(63.42%)
	No, uninsured	739(36.58%)
Vaccinations		
Willingness to be vaccinated against MPOX	Yes, Willing	652(31.93%)
	No, unwilling	872(42.70%)
	None respondents	518(25.37%)
Vaccinated in the past	Yes	624(30.60%)
	No	1,337(65.57%)
	Prefer not to say	78(3.83%)
Do you know about vaccines for MPOX?	Yes	74(9.05%)
	No	744(90.95%)
MPOX Knowledge		
Continuous variables		
Household size	Observation (Mean, SD)	1,980(5.93, 4.34)
Vaccine knowledge	Observation (Mean, SD)	808(15.28, 4.97)

SD represents the standard deviation.

### Adjusted and unadjusted logistic regression models with odd ratios and confidence intervals

Table 2 below presents three models. Models 1 and 2 present unadjusted odds ratios (UOR). Model 1 examines the relationship between self-rated health and willingness to accept the MPOX vaccine. Model 2 examines previous vaccine uptake and willingness to accept the MPOX vaccine. On the other hand, the third model (model 3) provides the adjusted odd ratios (AOR) of models 1 and 2. The adjusted variables are gender, age, employment status, health insurance status, and MPOX virus and vaccine knowledge.

**Table 2:** Bivariate and multivariate logistic regression of self-rated health and previous vaccine uptake on willingness to accept MPOX vaccine.

Variables		Model 1	Model 2	Model 3
		AOR (95% CI)	AOR (95% CI)	UOR (95% CI)
Self-rated health status	Excellent health	Ref		Ref
	Poor health	2.07(1.43–2.98) **		1.65(.87–3.11)
Previous vaccine uptake	Yes		Ref	Ref
	No		.48(.39-.60) **	.29(.19-.44)***
	Prefer not to say		.10(.04-.25) **	. 89 (. 07–10.61)
Gender	Male			Ref
	Female			1.65(1.13–2.40)**
Age	<30			Ref
	30–40			1.34(. 83–2.16)
	>40			2.25(1.40–3.62)**
Employment status	Employed			Ref
	Unemployed			1.60(1.07–2.41)*
Marital status	Single			Ref
	Married			1.59(1.06–2.39)*
Average monthly income	< ghc2000			Ref
	Ghc2001-ghc4000			.67(.37–1.22)
	>ghc 4000			.25(.14-.45)***
Valid health insurance	Yes			Ref
	No			1.29(.88–1.90)
Vaccine knowledge				.97(.93–1.00)
Household size				1.00(.95–1.06)
_cons		.70(.63-.78)	1.25(1.05–1.48)	2.11(.98–4.52)
Number of observations		1,524	1,517	594
LR chi2(1)		15.52	67.83	90.18

OR represents odds ratio, UOR represents unadjusted odd ratio and AOR represents adjusted odd ratio

* p<0.05,

**p<0.01,

***p<0.001

The results from model 1 indicate that self-rated health is significantly associated with the willingness to receive the MPOX vaccine. Compared to those who reported their health status as “Excellent,” individuals who reported their health status as “poor” (UOR 2.07; 95% CI: 1:.43–2.98) were significantly more willing to receive an MPOX vaccine. Similarly, model 2 shows a statistically significant association between previous vaccine uptake and the willingness to accept the MPOX vaccine. Compared to individuals who have previously accepted a vaccine, those who said no to previous vaccines (UOR .48; 95% CI: .39–.60) and those who prefer not to say (UOR .10; 95% CI: .04–.25) were significantly less likely to accept the MPOX vaccine.

Even after adjusting for covariates in model 3, there is still a significant link between vaccine uptake history and willingness to get the MPOX vaccine. Thus, people who have not been vaccinated in the past are much less likely to get the vaccine (AOR .29; 95% CI: .19–.44). In model 3, although there is a relationship between poor health and the willingness to accept the MPOX vaccine (AOR 1.65; 95% CI: .87–3.11), the relationship is not significant. Meaning there is an interaction between self-rated health and the covariates presented in model 3.

A significant association was found between gender and the willingness to accept the vaccine. Females were significantly more willing to accept the vaccine (AOR 1.65; 95% CI: 1.13–2.40) than their male counterparts. The results further indicate that compared to those less than 30 years old, those above 40 are significantly more willing (AOR 2.55; 95% CI: 1.40–3.62) to accept the MPOX vaccine. Interestingly, we found that compared to the employed, the unemployed are more willing to accept the vaccine (AOR 1.60; 95% CI:1.07–2.41). In addition, we found that compared to the unmarried, those married are more willing to accept the MPOX vaccine (AOR 1.59; 95% CI: 1.06–2.39). We also found that compared to those who earn less than GHC 2000 (the “poor”), those who earn more than GHC 4000 (the “rich”) are significantly (AOR .25; 95% CI: .14–.45) less willing to accept the MPOX vaccine. Regarding knowledge of the MPOX disease and vaccine, albeit not significant, we found that those with high vaccine knowledge are less willing to receive the MPOX vaccine.

## Discussion and conclusions

On July 23, 2022, MPOX was declared a Public Health Emergency of International Concern by the World Health Organization (WHO). Even though the vast majority of reported cases are outside of the WHO African region, the poor health systems in this context underscore the urgent need for research on the factors influencing MPOX vaccine uptake [20, 21]. The emergence and re-emergence of infectious diseases, many of which tend to be public health threats, further demonstrate the essential need for evidence-informed public health policies and measures to enhance vaccine uptake. In this study, we examine the associations between self-rated health, vaccine uptake history, and the willingness to accept the MPOX vaccine by adults in Ghana during a public health emergency. At the bivariate level, we observed that individuals who rated their health as poor were significantly more willing to receive the MPOX vaccine than their counterparts who rated their health as excellent. However, after adjusting for sociodemographic and economic variables and participants’ knowledge, this relationship disappeared, suggesting the critical role of these variables in mediating the relationship between self-rated health and the uptake of the MPOX vaccine [15, 22]. For example, sociodemographic factors such as age and gender are found to be associated with MPOX vaccine acceptance in Ghana [5] and Ethiopia [23].

Findings from this study are consistent with previous studies establishing that previous uptake of vaccines is critical to their future acceptance [5, 12, 13]. For instance, Ghazy and colleagues, using data obtained from individuals on social media platforms, observed among Ghanaians that those vaccinated against COVID-19 had higher MPOX vaccine acceptance than their counterparts who did not [5]. Winter and colleagues made similar observations in the United States [24]. The authors found the receipt of COVID-19 vaccine to predict MPOX vaccine acceptance strongly. Previous vaccination history was also significantly associated with MPOX vaccine acceptance among healthcare workers in Jordan [25]. In Shenzhen, China, past influenza vaccine receipt was significantly associated with MPOX vaccine acceptance among adults [26]. An earlier systematic review of the literature on the factors associated with vaccination against pandemic influenza found that individuals vaccinated earlier against seasonal influenza were more likely to be vaccinated against pandemic influenza [12]. Raut et al. [13] drew similar conclusions in a recent review. The acceptance of vaccines in the past suggests that these individuals acknowledge the critical role of vaccines in reducing mortality and hospitalization rates and are, hence, inclined to accept the MPOX vaccine [26, 27]. Moreover, it is plausible that individuals vaccinated in the past generally trust vaccines and their overall efficacy and safety and, hence, are likely to accept the MPOX vaccine [5, 25, 28]. Having people who previously received vaccines testify to the efficacy and safety of vaccines through public health communication and messaging will be helpful in promoting vaccine uptake.

We observed that individuals’ sociodemographic and economic status also influence their willingness to take the MPOX vaccine. Specifically, females were found to be more likely to accept the vaccine compared to their male counterparts. This finding is consistent with earlier studies on COVID-19, influenza, and MPOX [12, 22, 28–30]. For instance, Braimah et al. [29] argue that women tend to take less risk and are more health conscious than their male counterparts and, hence, may be more willing to accept the MPOX vaccine. However, this finding contrasts with earlier studies, contending that females are less likely to take MPOX and influenza vaccines in Ghana [5] and Hungary [31]. Participants’ age was also significantly associated with their willingness to accept the MPOX vaccine, with individuals over forty more likely to accept the vaccine than their counterparts under thirty. This finding is consistent with previous studies linking age to health-seeking behaviors, including the willingness to accept vaccines [15, 31]. Evidence shows that age is a risk factor for diseases [14, 22, 32], including infectious diseases, and hence, older individuals may be willing to accept vaccines to minimize these risks, possibly explaining our findings. Furthermore, we found unemployed people more willing to accept the vaccine than employed people. This finding is surprising as employed individuals tend to be more exposed to the virus, especially in the workplace. A qualitative exploration of how employment influences behaviors toward vaccination will be beneficial. Earlier studies suggest that married individuals are more likely to act against infectious diseases to protect their families [29, 33]. In line with these studies, married individuals are more likely to accept the MPOX vaccine than their unmarried counterparts.

Besides sociodemographic and economic factors, knowledge of the cause and transmission mode of MPOX was observed to influence people’s willingness to vaccinate against it. This finding is not surprising and aligns with earlier studies [14, 29, 31]. Knowledge of the source of infection for the MPOX disease will enhance people’s trust and confidence, which can positively influence their attitudes towards the vaccine and its acceptance [15, 26]. Accurate knowledge about diseases’ causes and transmission modes will also aid in better assessing their risks and associated behaviors. Thus, public health promotion measures for MPOX disease will also need to be tailored to enhance people’s knowledge about its causes, transmission, and risks.

This study is among the first to examine how self-rated health and vaccine uptake history influence people’s willingness to receive the MPOX vaccine during a public health emergency, drawing from a nationally representative sample. Thus, our findings are generalizable to similar contexts. The findings are instrumental in enhancing knowledge of vaccine hesitancy and providing policy directions for public health promotion. Nonetheless, several limitations exist that are worth noting. Firstly, the data for the study is cross-sectional, limiting the interpretation of our findings to statistical associations. Thus, longitudinal and qualitative studies will be useful in addressing this limitation. Furthermore, answers to our questions may be influenced by participants’ experiences with COVID-19 as our data were collected immediately after the height of the pandemic, subjecting our findings to potential bias. Finally, although we accounted for the role of sociodemographic factors in influencing the relationship between self-rated health and willingness to take the MPOX vaccine in model 3, we did not perform further interaction analysis, as that is beyond the scope of this paper. Thus, future studies could benefit from mediation/interaction analysis to unveil the specific sociodemographic and economic variables mediating the relationships between self-rated health, vaccine acceptance history, and willingness to receive the MPOX vaccine.

Notwithstanding these limitations, our findings demonstrate that people’s willingness to accept the MPOX vaccine may depend on multiple factors, including their vaccine uptake history, age, gender, income, and knowledge of the etiology of the disease. Based on these, we make some policy recommendations. First, we recommend a multi-pronged public health approach in addressing vaccine rejection or apathy in the Ghanaian context and perhaps other low- and middle-income countries. Thus, public health measures to enhance vaccine uptake should pay particular attention to people’s vaccine histories, given its association with the willingness to accept the MPOX vaccine. Second, we propose gender-sensitive public health policies and programs focusing on men with relatively higher vaccine hesitancy levels. Finally, we recommended involving community members with up-to-date vaccination history in vaccine-related public health education and programming. The educational programs should particularly focus on clarifying MPOX vaccine safety concerns.

## Supporting information

S1 Text Models. (DOCX)

S1 Fig ROC curve with associated AUC for model 3. (DOCX)
