# Supplementary material for: Do self-rated health and previous vaccine uptake influence the willingness to accept MPOX vaccine during a public health emergency of concern? A cross-sectional study
Source: PLOS Glob Public Health. 2024 Aug 15;4(8):e0003564. doi: 10.1371/journal.pgph.0003564 (PMC11326589; doi:10.1371/journal.pgph.0003564)
Supplement: S1 Text — (DOCX) [file pgph.0003564.s001.docx]

___ ____ ____ ____ ____ ®

/__ / ____/ / ____/ 18.0

___/ / /___/ / /___/ BE—Basic Edition

Statistics and Data Science Copyright 1985-2023 StataCor

> p LLC

StataCorp

4905 Lakeway Drive

College Station, Texas 77845

> USA

800-STATA-PC https://

> www.stata.com

979-696-4600 stata@st

> ata.com

**MODEL 1**

// model 1_ self-rated health and vaccine uptake

. logistic Vaccine_willingness1 i.Q12

Logistic regression Number of obs = 1,524

LR chi2(1) = 15.52

Prob > chi2 = 0.0001

Log likelihood = -1032.6634 Pseudo R2 = 0.0075

Vaccine_willingness1 Odds ratio Std. err. z P>z [95% conf. interval]

Self-rated health

Poor 2.069336 .386113 3.90 0.000 1.435512 2.983011

_cons .7020757 .0381979 6.50 0.000 .6310627 .7810797

Note: _cons estimates baseline odds.

.

end of do-file

**Model 2**

do "/var/folders/74/0_s3x1q94ysg5lky26bpjyw00000gn/T//SD08762.000000"

. //model_2 Previous vaccine uptake

. logistic Vaccine_willingness1 i.Q3

Logistic regression Number of obs = 1,517

LR chi2(2) = 67.83

Prob > chi2 = 0.0000

Log likelihood = -1000.8401 Pseudo R2 = 0.0328

Vaccine_willingness1 Odds ratio Std. err. z P>z [95% conf. interval]

Previous vaccine uptake

No .482361 .0539404 -6.52 0.000 .3874235 .6005627

Prefer not to say .1046293 .0463789 -5.09 0.000 .0438877 .2494391

_cons 1.246637 .1120686 2.45 0.014 1.045249 1.486826

Note: _cons estimates baseline odds.

.

end of do-file

**MODEL 3**

. do "/var/folders/74/0_s3x1q94ysg5lky26bpjyw00000gn/T//SD08762.000000"

. logistic Vaccine_willingness1 i. Self-rated health status i. Previous vaccine uptake i.Gender i.Age i.Employment_status i. Marital Status i.Average_monthly_income

> i. Valid health insurance Household size Vaccine_knowledge

Logistic regression Number of obs = 594

LR chi2(13) = 90.18

Prob > chi2 = 0.0000

Log likelihood = -348.00455 Pseudo R2 = 0.1147

Vaccine_willingness1 Odds ratio Std. err. z P>z [95% conf. interval]

Self-rated health

Poor 1.654955 .5347215 1.56 0.119 .8785368 3.117541

Previous vaccine uptake

No .2991449 .0616712 -5.85 0.000 .1997098 .4480885

Prefer not to say .8931835 1.127904 -0.09 0.929 .0751711 10.61282

Gender

female 1.655066 .3158191 2.64 0.008 1.138648 2.405699

Age

31-40 1.34703 .3265176 1.23 0.219 .8376213 2.166242

>40 2.258915 .5447634 3.38 0.001 1.408065 3.623906

Employment_status

Unemployed 1.608678 .3351534 2.28 0.022 1.069373 2.419966

Marital Status

Married 1.592521 .330431 2.24 0.025 1.0604 2.391664

Average_monthly_income

Ghc2001-ghc4000 .6780228 .2042926 -1.29 0.197 .3756399 1.223818

>ghc 4000 .2556935 .0747917 -4.66 0.000 .1441249 .4536288

Valid health insurance

No 1.298408 .2547143 1.33 0.183 .8839493 1.907195

Household size .9915634 .0260972 -0.32 0.748 .9417107 1.044055

Vaccine_knowledge .9715858 .0180697 -1.55 0.121 .9368075 1.007655

_cons 2.113668 .8217613 1.93 0.054 .9865127 4.52867

Note: _cons estimates baseline odds.

.

end of do-file

.
