# Supplementary figures and images for: Do self-rated health and previous vaccine uptake influence the willingness to accept MPOX vaccine during a public health emergency of concern? A cross-sectional study
Source: PLOS Glob Public Health. 2024 Aug 15;4(8):e0003564. doi: 10.1371/journal.pgph.0003564 (PMC11326589; doi:10.1371/journal.pgph.0003564)

ROC curve with associated AUC for model 3


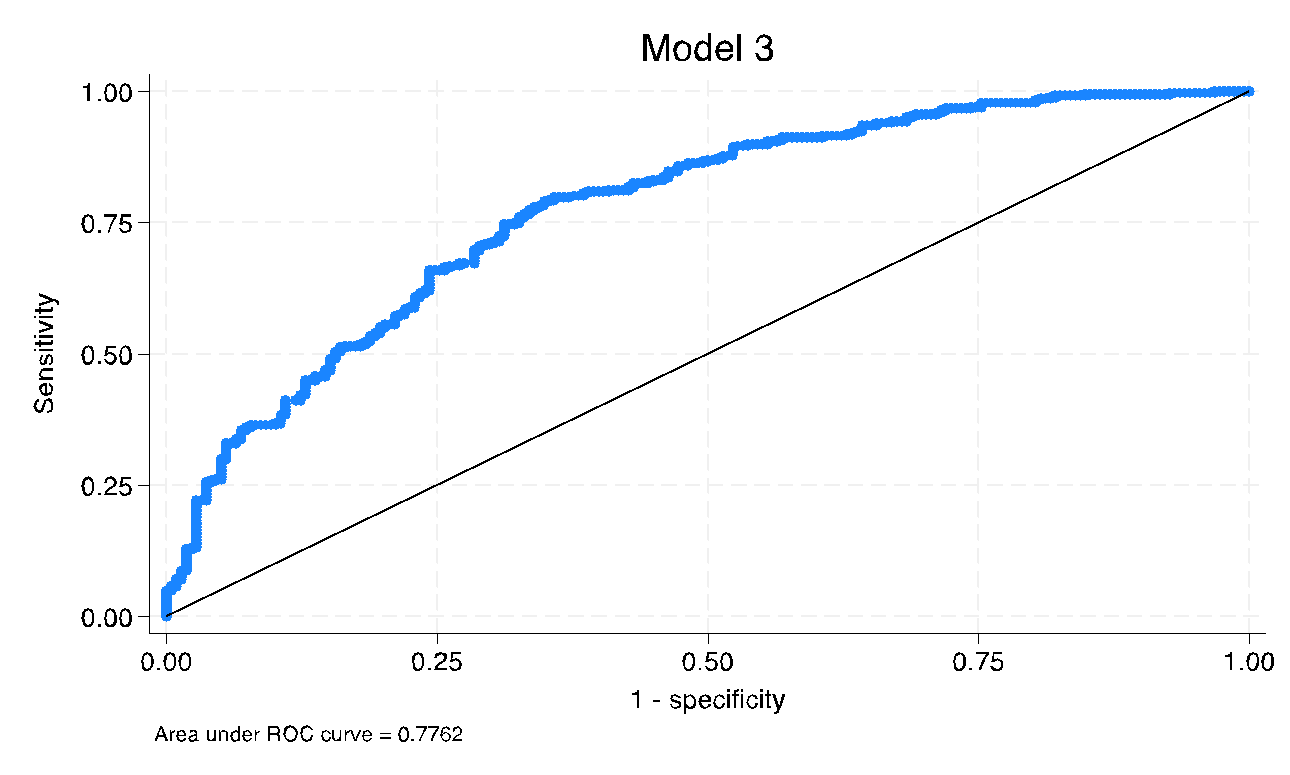

Supplement: S1 Fig — (DOCX) [file pgph.0003564.s002.docx]
